# Anatomical Reconstruction of Chronic Distal Biceps Tendon Ruptures Using a Tripled Semitendinosus Auto-Graft, Tension-Slide Technique and Interference Screw: Description of a New Surgical Technique and Preliminary Results

**DOI:** 10.3390/jcm14227948

**Published:** 2025-11-10

**Authors:** Ferdinando Maria Pulcinelli, Alessandro Caterini, Giuseppe Rovere, Matteo D’Ambrosio, Giacomo Maria Minnetti, Pasquale Farsetti, Fernando De Maio

**Affiliations:** Department of Clinical Sciences and Translational Medicine, Section of Orthopaedics and Traumatology, University of Rome Tor Vergata, 00133 Roma, Italy

**Keywords:** chronic distal biceps tendon rupture, tripled semitendinosus autograft, tension-slide technique, biceps button, tensiomyography

## Abstract

**Background:** The distal biceps brachii tendon inserts proximally and posteriorly on the bicipital tuberosity of the radius and it is a forearm supinator but also contributes to flexion of the elbow. Chronic distal biceps tendon ruptures are relatively rare, often complicated by tendon and muscle retraction, and, therefore, their primary repair is difficult or impossible. The gold standard treatment of these chronic lesions is its anatomic reinsertion at the radial tuberosity after tendon reconstruction, using autograft or allograft tissue, but there is no agreement about the most appropriate surgical technique. Untreated injuries usually result in elbow joint deficits and decreased muscular strength. We report the preliminary results in a group of patients treated with a tripled autologous semitendinosus graft. **Methods:** In the present retrospective study, we report the results in a series of 13 patients surgically treated using tripled autologous semitendinosus graft, fixed to the residual distal biceps tendon, starting from the myotendinous junction, and using tension-slide technique (Biceps Button—Arthrex, Inc, Naples, FL 34108, USA) in association with an interference screw. Eleven males and two females, with a mean age of 46, participated in the study. **Results:** At mean follow-up check-in of 35 months, clinical results were assessed using the DASH score and MEPS, with a mean value of 11 points and 87 points, respectively. Tensiomyography was also performed to evaluate muscular strength. Six patients had excellent results and seven had good results. No patient had either a tendon re-rupture, or a peripheral neurological deficit, or symptomatic heterotopic ossifications. Seven patients had a mild deficit in elbow motion and six patients had a mild deficit in forearm prono-supination. Upon tensiomyography evaluation, five patients showed a mild deficit in flexion and supination strength. All patients returned to their previous daily and sporting activities. **Conclusions:** According to our results, in patients affected by chronic distal biceps tendon rupture, surgical treatment performed using tripled autologous semitendinosus autograft secured to the radial tuberosity using the tension-slide technique and interference screw is associated with satisfactory outcomes.

## 1. Introduction

The distal biceps tendon shows two distinct heads, the long head and short head. The long head inserts proximally and posteriorly on the bicipital tuberosity of the radius. This head contributes mainly to the supination movement of the forearm. The short head inserts more distally on the radial tuberosity, giving it a greater elbow flexion moment [1]. For this reason, its anatomical reconstruction is essential to restore complete muscle and forearm function.

Rupture of the distal biceps brachii tendon often occurs after an eccentric loading of the dominant elbow in men aged 40 to 60, with an estimated incidence of 2.55 cases per 100,000 patients per year [2,3].

Typical symptoms of the rupture of the distal biceps tendon include edema, ecchymosis, proximal retraction of the tendon (reverse Popeye sign), and decreased strength in elbow flexion and in forearm supination. The most used test to confirm the diagnosis is the hook test [4]. Clinical exams are often sufficient to discover an acute rupture but the lesion is not always immediately diagnosed. In cases of doubt, MRI represents the imaging modality of choice.

Chronic distal biceps ruptures are defined as those persisting for over 3–6 weeks post injury [5,6,7]. The untreated injury usually results in elbow joint deficits and decreased muscular strength, especially in elbow flexion and forearm supination. In acute cases, tendon ruptures can be repaired primarily while, in chronic tears, musculo-tendinous retraction and tissue atrophy make a direct repair often impossible.

Some surgeons treat chronic tears of the distal biceps conservatively or surgically via direct reinsertion, or via tenodesis of the retracted tendon to the distal brachialis muscle. Conservative treatment is generally reserved for patients with severe comorbidities or those of very old age and with limited functional requirements. Patients conservatively treated, in fact, usually show a decreased supination and flexion strength of the elbow. Direct reinsertion is very difficult because of the retraction of the musculo-tendinous unit. Moreover, the excessive tension of the reinserted tendon exposes to a high risk of failure for high rate of re-rupture, or for a residual functional limitation of the forearm extension. Tenodesis of the retracted tendon on the distal brachialis muscle does not determine any limitation in the extension of the forearm, and it improves muscle strength in elbow flexion but does not improve muscle strength in forearm supination. Therefore, in distal biceps reconstruction, it is technically demanding to restore a good function of the forearm movement without significant limitations in daily life activities [8,9,10,11,12,13,14,15,16,17].

The most appropriate surgical option for distal biceps tendon reconstruction is still controversial; however, many authors agree that the gold standard treatment for chronic irreparable distal biceps tear is anatomic reinsertion with the additional use of an autograft or allograft tissue to restore the musculo-tendinous’ original length. Many graft options have been reported and the most common include the fascia lata autograft, tibialis anterior allograft, Achilles tendon allograft, lacertus fibrosus autograft, and semitendinosus auto and allograft [18,19,20,21,22,23].

In the present study, we reported the preliminary outcomes of 13 patients affected by chronic distal biceps ruptures who were treated via anatomical tendon reconstruction with a tripled autologous semitendinosus graft using tension-slide technique in association with an interference screw.

The triplication of the tendon graft guarantees a very good mechanical resistance of the reconstructed tendon. Moreover, the reinsertion on the radial tuberosity using the tension-slide technique and interference screw allows for an immediate stability to be achieved with the excellent adhesion of the graft into the radial bone tunnel.

## 2. Materials and Methods

### 2.1. Study Design and Sample

We conducted a retrospective study on a sample of 13 patients with chronic distal biceps brachii tendon rupture that was surgically treated with a tendon reconstruction using a triple autologous semitendinosus graft that was reinserted in the bicipital tuberosity via the tension-slide technique (Biceps Button—Arthrex, Inc., Naples, FL 34108, USA) and interference screw. The only inclusion criterion was a traumatic chronic rupture of the tendon persisting for over 3–6 weeks post injury. Eleven patients were males and two were females, with a mean age of 46 (range: 29–59 y). The right side was affected in ten cases and the left side in three. Patients with open traumatic injuries, metabolic pathologies, and those undergoing dialysis were excluded from the study. Written informed consent was obtained from all individual participants included in the study. All procedures performed in the study were in accordance with the 1964 Helsinki Declaration and its later amendments.

### 2.2. Surgical Technique

The harvest of the semitendinosus tendon is made through an incision of 3–4 cm at the antero-medial margin of the tibia.

**Figure 1:**
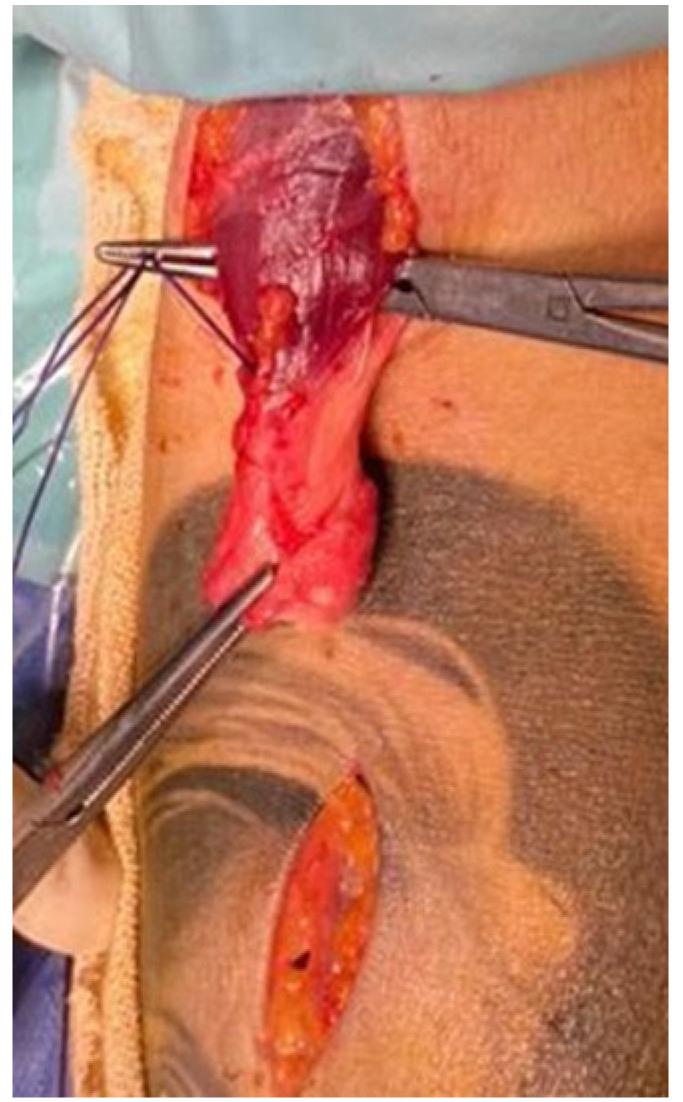
Two incisions are made; one is proximal, to locate the tendon stump, and one is distal, at the level of the bicipital tuberosity on the radius.

In the upper limb, an incision is centered on the radial tuberosity and another incision is made proximally, to search for the tendon stump (Figure 1).

**Figure 2:**
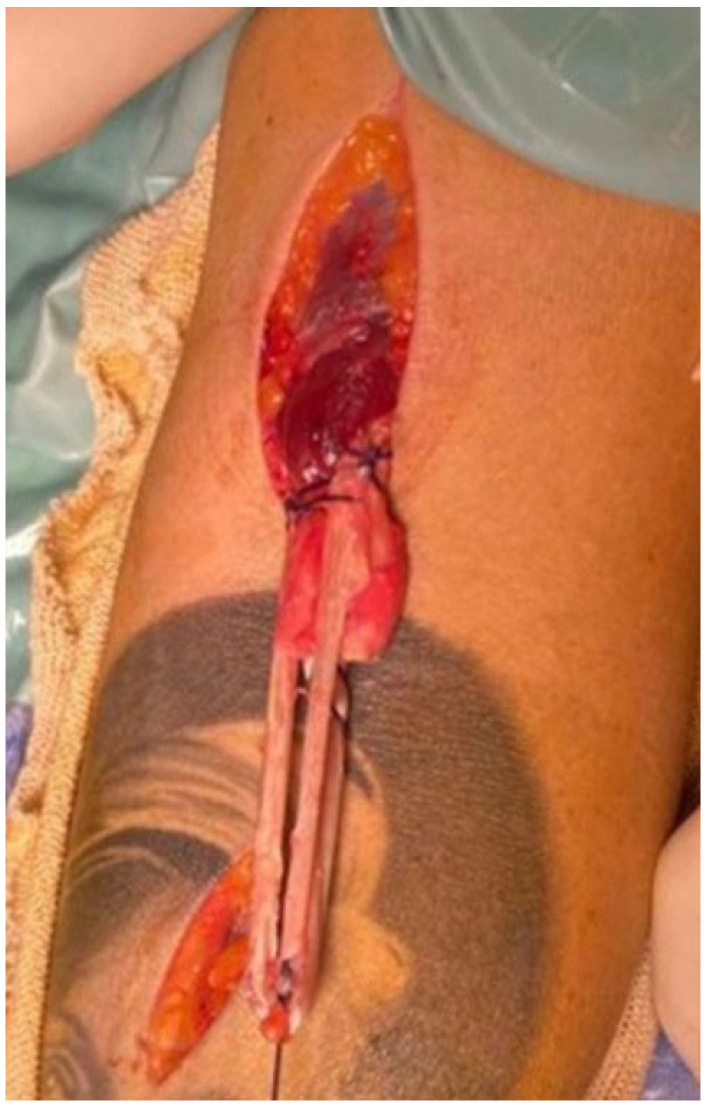
Passage of sutures through the myotendinous junction, in order to anchor the tripled semitendinosus graft to the residual distal biceps tendon.

To reconstruct the tendon, the semitendinosus is fixed to the residual distal biceps tendon, starting from the myotendinous junction, using the modified Pulvertaft method and tripling it, according to the technique already described for anterior cruciate ligament (ACL) reconstruction [24] (Figure 2).

**Figure 3:**
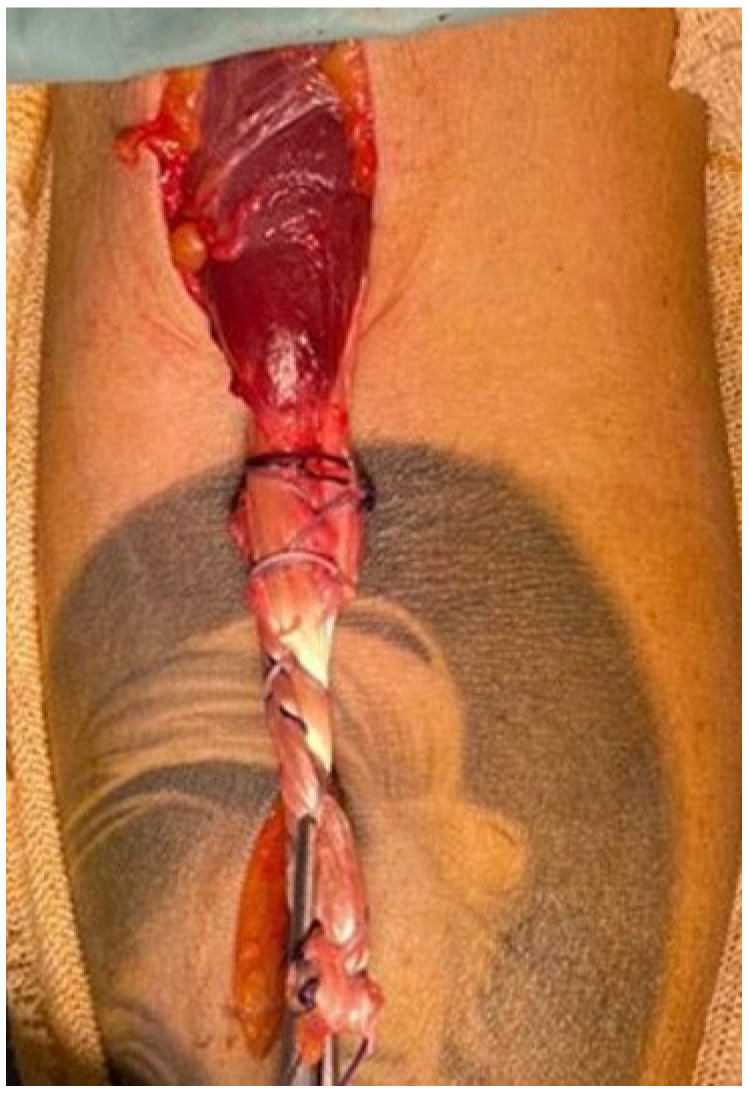
The final aspect of the tripled semitendinosus autograft anchored to the residual distal biceps tendon.

The reconstructed tendon (Figure 3) is passed through the subcutaneous tissue, under the skin, from the proximal approach to the distal one. Next, we drill the bone to pass the button, and a hemi-tunnel (with a diameter of 8 mm) is made in the biceps tuberosity to allow for tendon–bone healing. Using an insertion device, we pass the button through to the other side of the bone. Once in place, the button is flipped against the bone cortex and the sutures are tightened to secure the biceps tendon in the hemi-tunnel. For additional stability, we fix the tendon inside the hemi-tunnel with a peak interference screw.

In all cases, the reinsertion was performed at the radial tuberosity corresponding anatomically to the distal insertion of the biceps brachii tendon. We selected tripled semitendinosus autograft because it guarantees a very good mechanical resistance of the reconstructed tendon and eliminates the associated risks of allograft tissues or synthetic augmentation. Therefore, this autograft offers a satisfactory length, and the donor site morbidity is rare. The same team, particularly experienced in sports traumatology, performed all surgical procedures.

### 2.3. Post-Surgical Rehabilitation Phase

The upper limb was initially immobilized with a brace that locked the elbow at 90° of flexion and 45° of supination. After six weeks, the patients began the rehabilitation phase, starting with the passive movements of the pronation and supination of the forearm, while the active exercises of the extension and flexion of the elbow were permitted after about 8 weeks, without lifting even light weights. Three months after the surgery, the patients usually had full range of motion of the elbow and progressively began to perform muscular strength exercises to return to their previous activities after five months. The timing and progression of the rehabilitation phases were based on tissue biology to promote the initial integration of the graft into the bone radial tunnel. All patients adhered to the same rehabilitation protocol conducted or supervised by physiotherapists specialized in sports medicine.

### 2.4. Outcome Assessment and Classification

At the final follow-up check-in, on average 35 months after surgery, clinical results were assessed using the Disabilities of Arm, Shoulder, and Hand (DASH) score [25] and Majo Elbow Performance Score (MEPS) [26]. Radiographic examination of the elbow was also performed to exclude radio–ulnar synostosis or heterotopic ossifications. Furthermore, the patients were analyzed using tensiomyography (TMG-BMC Ltd.—1000 Ljubljana, Slovenia) to evaluate the muscle strength [27,28,29]. Clinical and instrumental outcome assessments were performed by independent evaluators. Tensiomyography is a non-invasive device for monitoring muscle contractile and mechanical characteristics via the analysis of some parameters. In our case, we analyzed, especially, the contraction time (Tc) and maximal radial displacement (Dm). The criteria used to categorize outcome classification as excellent, good, or poor were developed ad hoc.

The results were considered excellent if the DASH score ranged from 1 to 9 points, if the MEPS ranged from 90 to 100 points, and if, at clinical examination, the range of elbow motion, and flexion and supination strength, were similar to the contralateral side. The results were considered good if the DASH score ranged from 10 to 24 points, if the MEPS ranged from 75 to 89 points, and if the range of elbow motion and flexion and supination strength were mild, or limited in comparison to the contralateral side (about −15° of prono-supination and −15% of muscular strength). The results were considered poor if the DASH score was more than 24 points, if the MEPS ranged from 60 to 74 points, and if the range of elbow motion and muscular strength were, respectively, limited more than 30° and more than 15% in comparison to the contralateral side.

### 2.5. Statistical Analysis

All statistical analyses were performed using R software (version 4.x) and Jamovi (version 2.x). Continuous variables (age, time from injury to surgery, follow-up duration, DASH score, MEPS, joint range of motion, and tensiomyography parameters) were summarized as mean ± standard deviation (SD) with 95% confidence intervals (CI). Normality was checked using the Shapiro–Wilk test.

Categorical variables (sex, affected side, presence of range of motion deficit, and presence of tensiomyographic asymmetry ≥ 10%) were expressed as absolute frequencies and percentages.

Comparisons between groups were carried out with Welch’s two-sample *t*-test (unequal variances). Specifically, subgroup analyses were performed for the following:

1. Surgical timing (early ≤6 weeks vs. late ≥7 weeks);
2. Age (≤46 years vs. >46 years);
3. TMG asymmetry (absent <10% vs. present ≥10%).

The value of *p* < 0.05 (two-sided) was considered statistically significant. No adjustments for multiple testing were applied, given the exploratory nature of the study.

Patients were classified as having excellent, good, or poor outcomes, based on predefined thresholds combining DASH, MEPS, range of motion, and strength parameters. Descriptive statistics were used to report the distribution of patients across these categories.

## 3. Results

All patients had appropriate clinical and imaging documentation and were analyzed retrospectively at a mean follow-up of 35 months (range: 20–47 m).

Demographics, and the clinical and instrumental results of our patients are reported in Table 1, and statistical results are reported in Table 2.

No patient experienced tendon re-rupture and we did not observe any peripheral neurological deficits. Seven patients had a mild deficit in elbow motion, compared to the contralateral side, ranging from −10° to −15°, and six patients had a mild deficit in forearm prono-supination, ranging from −10° to −20°. No patient had any symptomatic heterotopic ossifications or radio–ulnar synostosis. Upon tensiomyography evaluation of the biceps brachii muscle, five patients exhibited a mild deficit in flexion and supination strength, ranging from −10% to −15% compared to the contralateral side. DASH score ranged from 5 to 16 points (mean value: 11 points), and MEPS ranged from 76 to 94 points (mean value: 87 points).

According to our classification criteria, six patients had an excellent result and seven a good result.

### Statistical Results

At the final follow-up, the mean DASH score was 10.7 ± 3.9 (95% CI 8.3–13.1) and the mean MEPS was 87.4 ± 5.7 (95% CI 83.9–90.8).

When comparing surgical timing, patients treated within six weeks from injury (*n* = 7) achieved significantly better results than those treated later (*n* = 6). Specifically, the early group reported lower DASH scores (8.8 ± 2.5 vs. 12.8 ± 3.7, t = –2.63, *p* = 0.028) and higher MEPS values (91.7 ± 2.1 vs. 82.5 ± 5.2, t = 4.39, *p* = 0.004).

With respect to age, younger patients (≤46 years, *n* = 7) showed superior functional outcomes compared with older patients (>46 years, *n* = 6). The younger group presented significantly lower DASH scores (7.9 ± 2.3 vs. 13.7 ± 1.8, t = –5.37, *p* < 0.001) and higher MEPS values (92.3 ± 2.1 vs. 81.7 ± 4.0, t = 6.36, *p* < 0.001).

Tensiomyography analysis (TMG) revealed that patients without relevant asymmetry (<10%, *n* = 8) had markedly better outcomes compared with those showing asymmetry ≥ 10% (*n* = 5). DASH scores were significantly lower (8.3 ± 2.7 vs. 14.8 ± 2.6, t = –4.18, *p* = 0.002), while MEPS values were significantly higher (91.8 ± 2.5 vs. 80.4 ± 4.6, t = 5.26, *p* = 0.002).

In summary, early surgical reconstruction, younger age, and preserved muscle contractility (absence of TMG asymmetry) were each associated with significantly better functional outcomes. We observed correspondence between clinical and instrumental findings regarding outcome assessment and classification. In fact, in patients with excellent results, range of motion and tensiomyography muscular strength of the operated limb were similar to those of the of the contralateral side. The patients with good results, instead, showed mild deficits in these clinical and instrumental parameters compared to the healthy side.

Finally, all patients were satisfied and returned to their previous daily and sporting activities, and the technique used for tendon reconstruction allowed for satisfactory functional recovery with low complication rates.

## 4. Discussion

A complete tear of the distal biceps brachii tendon is a relatively rare injury and generally occurs in middle-aged men, as a result of an eccentric contraction of the biceps muscle. This lesion can lead to a decrease in supination strength by about 50% and flexion strength by about 30% [20]. Chronic distal biceps ruptures, which are defined as those persisting for over 3–6 weeks post-injury, are usually complicated by tendinous retraction and scar tissue formation. The diagnosis of distal biceps tendon rupture is quite simple in acute lesions and generally is only based on a clinical evaluation. In chronic ruptures, the diagnosis is often delayed, MRI represents the imaging modality of choice, and their direct repair is a big challenge [17,23].

In elderly patients, especially those with comorbidities, conservative treatment is generally preferred. In healthy elderly patients, with low functional demands, a surgical alternative may be represented, such as the tenodesis of the biceps brachii tendon on the brachialis anterior, which improves muscle strength in elbow flexion but does not improve muscle strength in forearm supination.

The direct repair of complete ruptures of the distal biceps brachii tendon should be the treatment of choice. In acute ruptures, anatomical reinsertion on the radial tuberosity is easier because the tendon retains its original length. Chronic ruptures, on the contrary, are complicated by muscle–tendon retraction and tissue atrophy that make primary anatomic repair very difficult or impossible. In these cases, to restore original tendon length and muscle strength, surgical treatment is generally performed by a reconstruction of the musculo-tendinous unit with an interposition of autografts or allografts, and various graft options have been reported [7,8,9,10,11,12,13,14,15,16,30].

Morrel et al. reported good subjective and objective outcomes regarding the reconstruction of chronic ruptures using a fascia lata autograft secured to the bicipital tuberosity with suture anchors [10]. Cross et al. reported the outcomes in seven males who underwent biceps tendon reconstruction using tibialis anterior allograft [11]. The average time from injury to surgery was 25 weeks and the average follow-up was 16 months. The authors reported satisfactory clinical results with limited complications. One patient developed transient lateral antebrachial cutaneous nerve neuritis (LABNC). Phadnis et al. reported the results in 21 male patients affected by irreparable distal biceps ruptures treated using an Achilles tendon allograft and transosseous EndoButton [12]. After a mean follow-up of 15 months, the authors stated that all patients were satisfied and returned to their previous level of activity. Two transient LABNC paresthesia were recorded and two patients had a mild extension lag. Fontana et al. described the use of autologous lacertus fibrosus as augmentation–elongation technique in four patients affected by a retracted–degenerated distal biceps tendon [13]. Two patients had transient sensitive radial nerve paresthesia. All patients were reviewed at a mean follow-up of two years. The authors concluded that their surgical technique is an effective option for treatment of this chronic injury.

In our case series, we opted for a free autologous semitendinosus tendon to augment the muscle–tendon retraction when repairing chronic ruptures, as other authors have already described [8,9,14,15].

Hang et al. reported satisfactory results in a patient treated using a free semitendinosus autograft reattached to the radial tuberosity via a 2-incision technique [8]. Wiley et al. compared two groups of patients with chronic tendon ruptures [9]. The first group was treated non-operatively, while, in the second group, a reconstruction was performed using a semitendinosus autograft woven through the distal stump of the biceps tendon, then anchored into prepared radial tuberosity. After a mean follow-up of 28 months, the non-operated group showed a decreased muscular strength of about 20% while, in the operated group, muscular flexion and supination strength were similar to the contralateral side.

Frank et al. reported good outcomes in a series of 19 patients treated using a semitendinosus autograft looped through a transosseous tunnel in the bicipital tuberosity, and secured with a Pulvertaft weave to the remnant distal biceps tendon. They concluded that, when it is possible, direct repair is preferred, but in irreparable ruptures, reconstruction using semitendinosus allows surgeons to obtain good results [14].

Goyal et al. reported clinical and instrumental results in two groups of patients surgically treated for chronic ruptures using an allograft tendon looped around an EndoButton or using a 2-incision technique [15]. The semitendinosus allograft was woven into the native distal biceps tendon and secured with no. 2 FiberWire suture to reinforce the reconstruction. The authors evaluated the peak strength on both arms of the patients after a mean period of 46 months after surgery using an isokinetic dynamometry, and reported satisfactory results, but they observed decreased peak supination strength in the operated arm.

However, the most appropriate surgical option for distal biceps tendon reconstruction is still controversial. Many authors agree that the gold standard treatment for a chronic irreparable distal biceps tear is to restore the original tendon length and muscular strength through its anatomic reinsertion on the radial tuberosity and through tendon reconstruction with the additional use of an autograft or allograft tissue. The common grafts used are the fascia lata autograft, tibialis anterior allograft, Achilles tendon allograft, lacertus fibrosus autograft, and semitendinosus auto and allograft, but in all surgical options described, many complication rates are often reported. The most common complications reported are non-traumatic re-rupture, posterior interosseous nerve (PIN) palsy or lateral antebrachial cutaneous nerve (LABNC) paresthesia, heterotopic ossification, and elbow and forearm range of motion deficit [18,19,20,21,22,23].

Our results are similar to those reported by the above-mentioned authors, who used an autologous semitendinosus graft. Two recent systematic reviews support this technique, and the authors conclude that functional results are generally satisfactory after an allograft or autograft reconstruction [7,31].

In the majority of the reported studies, results are evaluated only by clinical tests and follow-up is often not reported. In our study, we performed clinical and instrumental tests using tensiomyography to evaluate the preliminary results of our surgical reconstruction technique. All patients, at follow-up, showed excellent or good results. We never observed a tendon re-rupture or neurological peripheral deficit. No evidence of heterotopic ossification was observed. No patients had a significant deficit in elbow motion or in forearm supination. Tensiomyography tests showed a mild deficit in flexion and supination strength in five patients. All patients were satisfied with the treatment outcome. Our preliminary results, although derived from a small retrospective case series, nevertheless suggest that the described technique, which involves the use of the tripled autologous semitendinosus tendon, reduces the risk of the recurrence of the rupture and allows almost complete recovery of strength in both flexion and supination. Finally, we agree that the anatomical reinsertion of the reconstructed tendon is necessary to improve movement and prevent heterotopic ossifications.

## 5. Conclusions

We conclude that in chronic irreparable distal biceps tendon ruptures, surgical reconstruction using the tripled autologous semitendinosus tendon, anchored in the bicipital tuberosity with the biceps button and interference screw, represents a valid treatment option. In fact, considering the low complication rate and the preliminary results, the new surgical technique described seems to be a safe procedure for the treatment of these chronic lesions. However, our study has two limitations: first, it is a retrospective study without any control group, and, second, the number of cases is quite limited. Further studies are necessary to confirm our preliminary outcomes.

## Figures and Tables

**Table 1:** Demographic clinical and instrumental results of 13 patients with chronic distal biceps tendon rupture surgically treated via anatomical reinsertion after tendon reconstruction using tripled autologous semitendinosus graft.

Case	Gender	Age	Side	Time from Injury to Surgery (Week)	Follow-Up (Months)	Elbow ROM Compared to Other Side	Forearm ROM Compared to Other Side	Tensiomyography Muscular Strength Asymmetries	DASH Score	MEPS	Results
1	M	39	R	5	40	NO DIFF	NO DIFF	NO DIFF	6	92	EXCELL
2	M	51	R	7	29	−15 EXT	−20 SUP −10 PRON	−15%	16	84	GOOD
3	M	43	R	5	32	NO DIFF	NO DIFF	NO DIFF	8	92	EXCELL
4	M	38	L	6	47	NO DIFF	NO DIFF	NO DIFF	7	90	EXCELL
5	F	41	R	4	28	NO DIFF	NO DIFF	NO DIFF	9	92	EXCELL
6	M	49	R	4	33	−10 EXT	NO DIFF	NO DIFF	12	86	GOOD
7	M	52	R	8	35	−10 EXT	−15 SUP −10 PRON	−15%	10	86	GOOD
8	M	59	L	9	41	−10 FLEX	−20 SUP −15 PRON	−15%	15	76	GOOD
9	F	43	L	5	37	NO DIFF	NO DIFF	NO DIFF	8	94	EXCELL
10	M	48	R	7	20	−15 EXT	−15 SUP −15 PRON	−10.0%	12	86	GOOD
11	M	54	R	10	30	−10 FLEX	−20 SUP −15 PRON	NO DIFF	15	78	GOOD
12	M	28	R	5	36	NO DIFF	NO DIFF	NO DIFF	5	94	EXCELL
13	M	50	R	7	46	−10 EXT	−15 SUP −15 PRON	−10.0%	16	86	GOOD

**Table 2:** Comparison of functional outcomes (DASH and MEPS) according to surgical timing, age, and TMG asymmetry (Welch *t*-test).

Group Comparison	Subgroup	DASH (Mean ± SD)	MEPS (Mean ± SD)	t-Value	*p*-Value
Surgery timing	Early (≤6 weeks, *n* = 7)	8.8 ± 2.5	91.7 ± 2.1	t = −2.63/4.39	*p* = 0.028/0.004
Surgery timing	Late (≥7 weeks, *n* = 6)	12.8 ± 3.7	82.5 ± 5.2		
Age group	≤46 years (*n* = 7)	7.9 ± 2.3	92.3 ± 2.1	t = −5.37/6.36	*p* < 0.001/<0.001
Age group	>46 years (*n* = 6)	13.7 ± 1.8	81.7 ± 4.0		
TMG asymmetry	Absent (<10%, *n* = 8)	8.3 ± 2.7	91.8 ± 2.5	t = −4.18/5.26	*p* = 0.002/0.002
TMG asymmetry	Present (≥10%, *n* = 5)	14.8 ± 2.6	80.4 ± 4.6		

Values are expressed as mean ± standard deviation (SD). Functional outcomes were assessed via the Disabilities of the Arm, Shoulder and Hand (DASH) score and the Mayo Elbow Performance Score (MEPS). Welch’s two-sample *t*-test was used; significance was set at *p* < 0.05.

## Data Availability

The datasets generated and/or analyzed during the current study are available from the corresponding author upon reasonable request.
